# Supplementary material for: Predicting visual function by interpreting a neuronal wiring diagram
Source: Nature. 2024 Oct 2;634(8032):113–23. doi: 10.1038/s41586-024-07953-5 (PMC11446822; doi:10.1038/s41586-024-07953-5)
Supplement: Supplementary file 6 — CRF and ERF predictions for individual TmY4 and TmY9 cells. Analogous to Supplementary Data 3, but for TmY target types. Shown are the top four monosynaptic pathways, the strongest pathway passing through each of the top ten intermediary types (ranking from Extended Data Fig. 7), and the trisynaptic pathway Tm1–TmY–Dm3–TmY (see the section entitled Prediction of spatial normalization). [file 41586_2024_7953_MOESM6_ESM.zip › DataS4/TmY9qΓèÑ/720575940638019930.pdf]

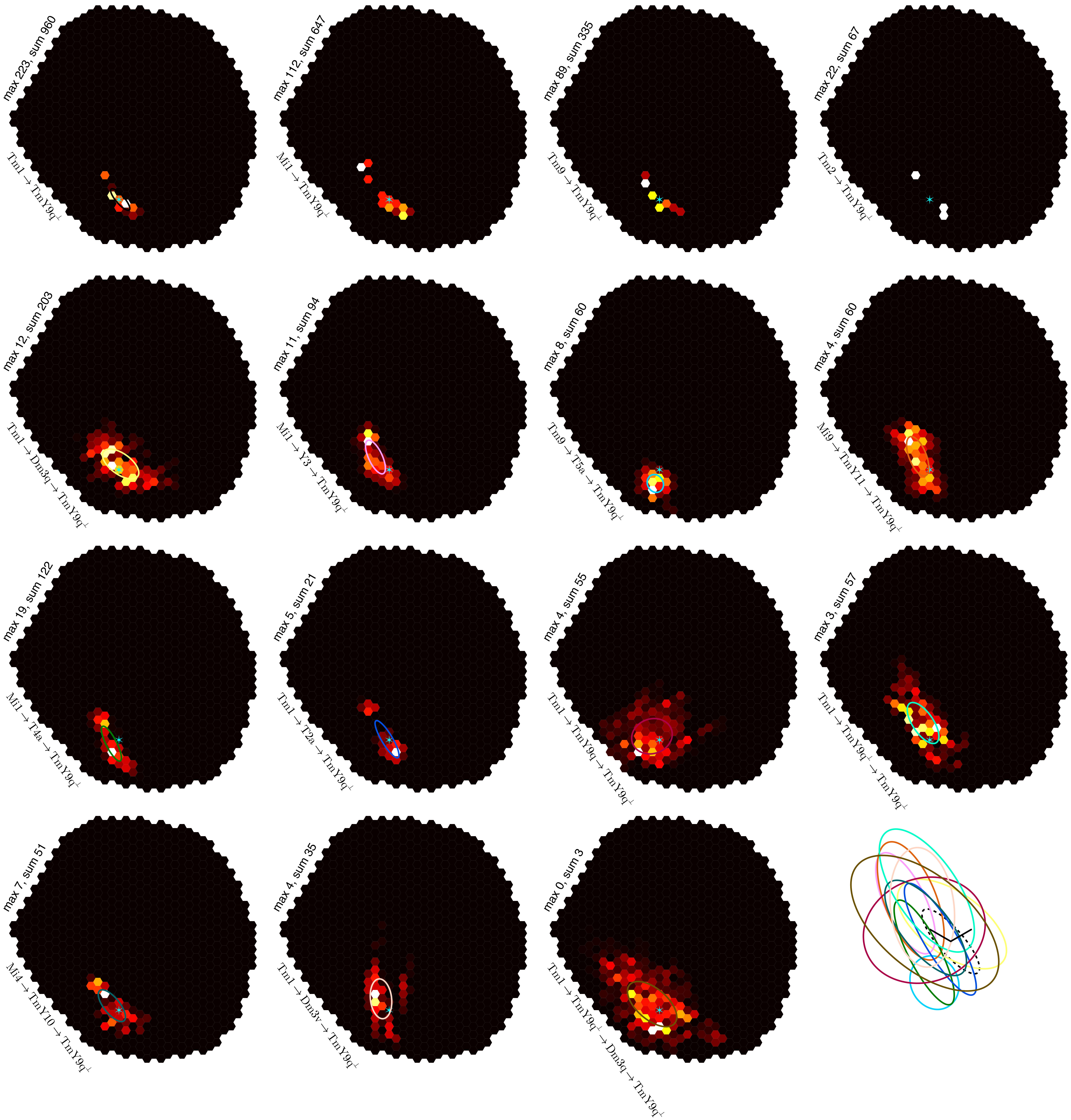

max 223, sum 960
Tm1 → TmY9q⊥
max 112, sum 647
Mi1 → TmY9q⊥
max 89, sum 335
Tm9 → TmY9q⊥
max 22, sum 67
Tm2 → TmY9q⊥
max 12, sum 203
Tm1 → Dm3q → TmY9q⊥
max 11, sum 94
Mi1 → Y3 → TmY9q⊥
max 8, sum 60
Tm9 → T5a → TmY9q⊥
max 4, sum 60
Mi9 → TmY11 → TmY9q⊥
max 19, sum 122
Mi1 → T4a → TmY9q⊥
max 5, sum 21
Tm1 → T2a → TmY9q⊥
max 4, sum 55
Tm1 → TmY9q → TmY9q⊥
max 3, sum 57
Tm1 → TmY9q⊥ → TmY9q⊥
max 7, sum 51
Mi4 → TmY10 → TmY9q⊥
max 4, sum 35
Tm1 → Dm3v → TmY9q⊥
max 0, sum 3
Tm1 → TmY9q⊥ → Dm3q → TmY9q⊥
